# Detecting type 2 diabetes mellitus cognitive impairment using whole-brain functional connectivity

**DOI:** 10.1038/s41598-023-28163-5

**Published:** 2023-03-09

**Authors:** Jinjian Wu, Yuqi Fang, Xin Tan, Shangyu Kang, Xiaomei Yue, Yawen Rao, Haoming Huang, Mingxia Liu, Shijun Qiu, Pew-Thian Yap

**Affiliations:** 1 The First School of Clinical Medicine, Guangzhou University of Chinese Medicine, Guangzhou, Guangdong China; 2 Department of Radiology and Biomedical Research Imaging Center, University of North Carolina, Chapel Hill, NC USA; 3 Department of Radiology, The First Affiliated Hospital of Guangzhou University of Chinese Medicine, Guangzhou, Guangdong China

**Keywords:** Neuroscience, Endocrinology, Medical research, Risk factors

## Abstract

Type 2 diabetes mellitus (T2DM) is closely linked to cognitive decline and alterations in brain structure and function. Resting-state functional magnetic resonance imaging (rs-fMRI) is used to diagnose neurodegenerative diseases, such as cognitive impairment (CI), Alzheimer’s disease (AD), and vascular dementia (VaD). However, whether the functional connectivity (FC) of patients with T2DM and mild cognitive impairment (T2DM-MCI) is conducive to early diagnosis remains unclear. To answer this question, we analyzed the rs-fMRI data of 37 patients with T2DM and mild cognitive impairment (T2DM-MCI), 93 patients with T2DM but no cognitive impairment (T2DM-NCI), and 69 normal controls (NC). We achieved an accuracy of 87.91% in T2DM-MCI versus T2DM-NCI classification and 80% in T2DM-NCI versus NC classification using the XGBoost model. The thalamus, angular, caudate nucleus, and paracentral lobule contributed most to the classification outcome. Our findings provide valuable knowledge to classify and predict T2DM-related CI, can help with early clinical diagnosis of T2DM-MCI, and provide a basis for future studies.

## Introduction

Type 2 diabetes mellitus (T2DM), accounting for the highest percentage of adults with diabetes, is a series of chronic endocrine and metabolic abnormalities. T2DM is related to clinical complications such as cognitive impairment (CI) and dementia. T2DM patients are at a 1.5 times higher risk for dementia or cognitive decline than individuals without diabetes^1,2^. Patients with diabetes manifest brain atrophy and microvascular disease in magnetic resonance imaging (MRI) exams^3^. However, factors contributing to the onset and progression of brain complications in patients with T2DM remain unclear. Therefore, there is an urgent need to identify these factors and early detection as the prevalence of T2DM is rising with population aging.

Increasing evidence indicates that patients with T2DM present structural and functional brain pathological changes^4^. In China, the prevalence of mild cognitive impairment (MCI), the prodromal stage of Alzheimer’s disease (AD), is 45% (ranges from 21.8 to 67.5%) in older patients with T2DM, substantially higher than 14.71% in older populations without T2DM. Prevalence is higher in older women^5–7^. MCI can gradually develop into moderate or severe CI and even AD^5^. Since AD cannot be completely cured, early detection and pharmacological and behavioral interventions of MCI are crucial for reducing the risk for AD^8^.

In the past 20 years, high-precision brain imaging techniques, such as structural, functional, and diffusion MRI as well as positron emission tomography (PET), have been demonstrated to be effective for investigating brain changes in patients with T2DM and MCI (T2DM-MCI)^9^. For instance, the blood oxygen level-dependent (BOLD) signal in fMRI, which reveals hemodynamic changes associated with neural activities, has been used to detect altered functional connectivity (FC) in patients with T2DM-MCI^5,10–12^. Diffusion tensor imaging (DTI), which quantifies the diffusion anisotropy of water molecules in white matter (WM), has been used to explore disruptions of structural network connectivity^13–16^. PET has been used to capture metabolic changes in the brain for early diagnosis^17^.

FC can be employed to reflect the functional condition of the brain, diagnose neurodegenerative diseases, and provide in-depth insights into pathophysiological mechanisms^8,18–20^. Region-specific FC provides useful features for T2DM-MCI classification. However, existing T2DM-MCI classification methods based on FC have limited accuracy of less than 70.0%^21,22^. Therefore, further effort is needed to improve the specificity and accuracy. The eXtreme Gradient Boosting (XGBoost)^23,24^, which improves classification based on iterative learning of weak classifiers. On a single machine, XGBoost is more than tenfold faster than existing popular solutions, with scalability to handle billions of samples. Model learning can be accelerated with parallel and distributed computing^23^. XGBoost is used in various applications^25,26^ owing to its high efficiency and accuracy. However, no previous study has used XGBoost for classifying patients with T2DM-MCI based on FC features. Therefore, the aim of the present study was to identify meaningful features to specifically distinguish T2DM-MCI.

Based on previous evidence, this study focused on the following objectives: (1) implement an efficient XGBoost classifier for T2DM-MCI classification; and (2) determine brain regions that distinguish patients with T2DM-MCI, providing a basis for early clinical diagnosis and interventional treatments.

## Results

### Clinical and neuropsychological results

A total of 199 participants underwent MRI, clinical blood, and neuropsychological scale tests and fulfilled the inclusion criteria. The mean ages at scanning for the T2DM-MCI, T2DM-NCI, and NC groups were 46.17 ± 8.67, 50.78 ± 8.28, and 46.30 ± 10.40 years, respectively. The demographic, clinical, and neuropsychological characteristics of the 199 participants are summarized in Tables 1,2,3. There were no significant differences between the T2DM-MCI, T2DM-NCI, and NC groups in sex, age, or educational level (*p* > 0.05). There were no statistically significant differences between the T2DM-MCI and T2DM-NCI groups in glycated hemoglobin levels, body mass index (BMI), or fasting blood glucose levels(*p* > 0.05). BMI was significantly different between the T2DM-NCI and NC groups (*p* = 0.001). Compared with the T2DM-MCI group, the T2DM-NCI and NC groups had higher levels of auditory verbal learning test (AVLT, immediate: *p* = 0.002 and *p* = 0.003; 5 min: *p* = 0.000 and *p* = 0.000; delay: *p* = 0.000 and *p* = 0.000; recall: *p* = 0.000 and *p* = 0.000), digit span test (reverse, *p* = 0.000 and *p* = 0.001), Montreal Cognitive Assessment-B (MoCA-B, *p* = 0.000 and *p* = 0.000), digit symbol substitution (DSST, *p* = 0.000 and *p* = 0.000), and lower levels of grooved pegboard test (GPT, L: *p* = 0.005 and *p* = 0.007; R: *p* = 0.000 and *p* = 0.000). There were no statistically significant differences in the other neuropsychological test outcomes among the three groups (*p* > 0.05) (Tables 1,2,3).

**Table 1:** Demographic results of T2DM-MCI, T2DM-NCI and NC groups.

	T2DM-NCI		T2DM-MCI		NC		*p*
	Mean	*SD*	Mean	*SD*	Mean	*SD*	
Age (years)	46.17	8.67	50.78	8.28	46.3	10.403	0.058
Gender (M/F)	60/33		23/14		43/26		0.969^#^
Educational level (years)	12.10	3.45	11.27	3.90	12.05	3.47	0.762
BMI (kg/m^2^)	24.70	2.93	23.43	3.12	23.05	2.76	0.003*

Data are shown as mean ± standard deviation (SD) and were analyzed using independent sample t-tests.

*BMI* body mass index.

^#^Pearson’s Chi-square test (2-sided).

*Statistically significant different (*p* < 0.05).

**Table 2:** Neuropsychological results of T2DM-MCI, T2DM-NCI and NC groups.

	T2DM-NCI		T2DM-MCI		NC		*p*
	Mean	*SD*	Mean	*SD*	Mean	*SD*	
AVLT (immediate)	23.71	4.92	20.41	5.51	23.84	5.15	0.008*
AVLT (immediate)	23.71	4.92	20.41	5.51	23.84	5.15	0.008*
AVLT (5 min)	9.46	2.27	7.50	2.67	10.09	4.11	0.000*
AVLT (delay)	9.14	2.46	6.97	2.96	9.28	2.23	0.000*
AVLT (recall)	11.07	2.10	8.21	3.97	11.03	1.88	0.000*
TMT-A	47.05	23.25	59.68	19.59	51.25	21.75	0.000*
TMT-B	40.18	17.01	48.76	15.87	43.23	16.72	0.009*
DST (direct)	7.85	1.47	7.32	1.61	10.08	9.16	0.102
DST (reverse)	4.95	1.26	3.89	1.59	5.10	1.64	0.000*
MoCA-B	27.51	1.55	24.05	0.85	27.52	1.54	0.000*
MMSE	28.49	1.54	27.83	1.84	28.32	1.69	0.170
DSST	49.75	12.89	35.03	12.77	48.24	14.83	0.000*
GPT (R)	74.17	13.53	90.70	27.53	68.49	14.17	0.000*
GPT (L)	81.60	15.25	93.81	30.89	80.10	18.22	0.023*

Data are shown as mean ± standard deviation (SD) and were analyzed using independent sample t-tests.

*AVLT* California-Los Angeles auditory verbal learning test, *TMT* trail-making test, *DST* digit span test, *MoCA* montreal cognitive assessment, *MMSE* mini-mental state examination, *DSST* digit symbol substitution, *GPT* grooved pegboard test, *L* left, *R* right.

*Data was considered significantly different (*p* < 0.05).

**Table 3:** Clinical results of T2DM-MCI, T2DM-NCI and NC groups.

	T2DM-NCI		T2DM-MCI		NC		*p*
	Mean	*SD*	Mean	*SD*	Mean	*SD*	
HbAlc (%)	9.25	2.56	8.75	2.15	NA	NA	0.522
DBP (mmHg)	126.42	16.62	127.79	19.96	123.48	19.04	0.273
SBP (mmHg)	82.74	14.00	82.25	10.72	82.40	11.12	0.783
FBG (mmol/L)	8.93	2.96	7.95	2.68	4.50	0.76	0.000*
FSI (uIU/mL)	11.25	9.79	14.08	16.05	NA	NA	0.507
TG (mmol/L)	2.67	2.58	3.25	3.83	NA	NA	0.775
TC (mmol/L)	4.76	1.03	4.99	1.06	NA	NA	0.694
LDL (mmol/L)	3.02	0.99	3.15	0.88	NA	NA	0.785
ACR (mg/g)	30.00	96.60	22.18	51.45	NA	NA	0.068
mALB (mg/L)	29.15	80.94	24.79	50.62	NA	NA	0.221
24 h UPRO (G/24 h)	0.19	0.23	0.17	0.14	NA	NA	0.436
M-TP (mg/L)	101.71	68.93	102.18	79.54	NA	NA	0.995
C-Peptide (ng/mL)	2.27	1.22	2.31	1.26	NA	NA	0.674

Data are shown as mean ± standard deviation, independent sample t-tests.

*Data was considered significant different (*p* < 0.05).

*HbA1c* hemoglobinA1c, *DBP* diastolic blood pressure, *SBP* systolic blood pressure, *FBG* fasting blood glucose, *FSI* fasting serum insulin, *TG* triglyceride, *TC* total cholesterol, *LDL* low-density lipoprotein, *ACR* albumin/creatinine ratio, *mALB* microalbuminuria, *24 h UPRO* 24-h urinary protein, *M-TP* micro total protein.

### Classification performance

A summary of classification performance using XGBoost is shown in Table 4 in terms of accuracy (ACC), the area under the curve (AUC), sensitivity (SEN), specificity (SPE), precision (PRE), and F1^27^. We constructed the XGBoost model to classify all three groups and pairwise classifications between two groups. The XGBoost model did not perform well in the three classification categories (ACC = 69.39%, AUC = 80.07%, SEN = 69.39%, SPE = 78.14%, PRE = 70.90%, and F1 = 68.11%); however, the model achieved peak performance in discriminating between the two classification categories (T2DM-NCI versus T2DM-MCI: ACC = 87.91%, AUC = 81.99%, SEN = 61.67%, SPE = 98.06%, PRE = 93.06%, and F1 = 73.95%; T2DM-NCI versus NC: ACC = 80.00%, AUC = 84.14%, SEN = 75.65%, SPE = 83.23%, PRE = 77.58%, and F1 = 76.24%).

**Table 4:** Classification performance in T2DM-MCI, T2DM-NC and NC differentiation.

	ACC (%)	AUC (%)	F1 (%)	SEN (%)	SPE (%)	PRE (%)
T2DM-NC vs. T2DM-MCI	87.91 ± 1.04	81.99 ± 8.30	73.95 ± 2.54	61.67 ± 4.56	98.06 ± 1.77	93.06 ± 6.36
T2DM-NC vs. NC	80.00 ± 0.83	84.14 ± 3.44	76.24 ± 1.48	75.65 ± 7.28	83.23 ± 6.20	77.58 ± 4.86
T2DM-NC vs. T2DM-MCI and NC	69.39 ± 1.27	80.07 ± 0.76	68.11 ± 0.87	69.39 ± 1.27	78.14 ± 1.33	70.90 ± 1.54

*ACC* accuracy, *AUC* the area under the receiver operating characteristic curve, *SEN* sensitivity, *SPE* specificity, *PRE* precision.

### Connections contributive to classification

**Figure 1:**
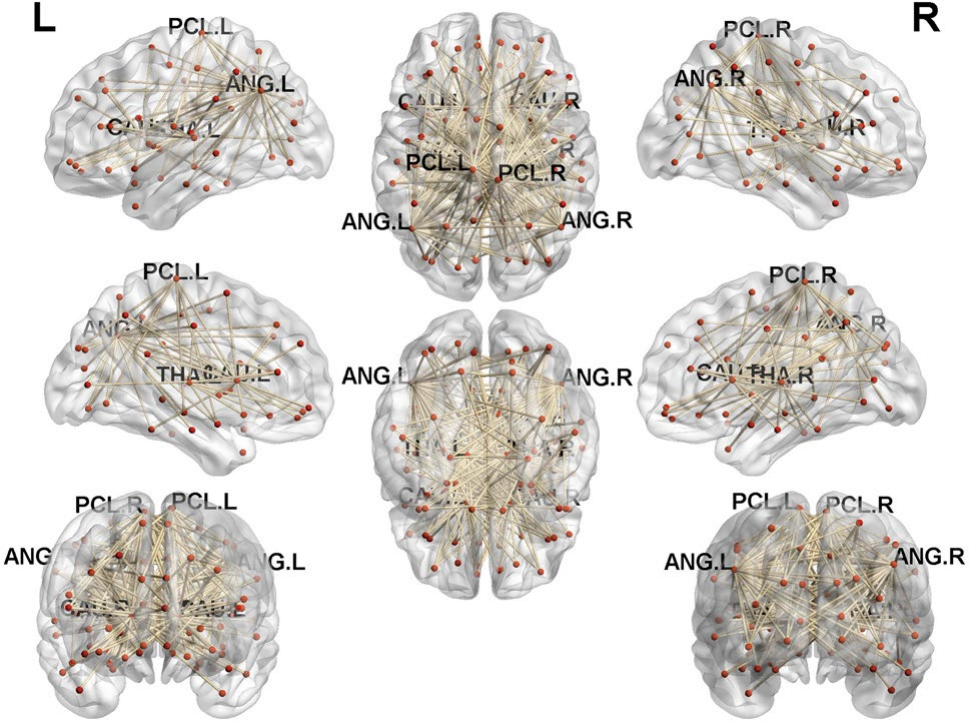
Significant connections rendered on the surface of the automated anatomical labeling atlas in BrainNet viewer. *THA* thalamus, *ANG* angular gyrus, *CAU* caudate nucleus, *PCL* paracentral lobule, *L* left, *R* right.

Out of 4,005 connections, 511 connections provided useful features for classification (analysis of variance (ANOVA) with Bonferroni correction, *p* < 0.05). After using the XGBoost model for classification, we traced the data to further explore and analyze the 511 functional connections. Many functional connections were connected through the same brain region; precisely, we observed the aggregation of connection features. We found that the following eight areas are most discriminative: left caudate nucleus (CAU.L, 14.68%), right caudate nucleus (CAU.R, 12.13%), left angular gyrus (ANG.L, 10.76%), left thalamus (THA.L, 7.83%), right paracentral lobule (PCL.R, 7.63%), right thalamus (THA.R, 7.44%), right angular gyrus (ANG.R, 6.65%), and left paracentral lobule (PCL.L, 4.7%). There were more than 70% of connections to CAU (26.81%), ANG (17.42%), THA (15.26%), and PCL (12.33%). The FC of these regions contribute most to T2DM-MCI classification (71.82% total, see Table 5 and Fig. 1).

**Table 5:** Brain regions most contributive to classification.

Brain region	Total ratio (%)	Subregion	Ratio (%)
CAU	26.81	CAU.L	14.68
		CAU.R	12.13
ANG	17.42	ANG.L	10.76
		ANG.R	6.65
THA	15.26	THA.L	7.83
		THA.R	7.44
PCL	12.33	PCL.L	4.70
		PCL.R	7.63

*CAU* caudate nucleus, *ANG* angular gyrus, *THA* thalamus gyrus, *PCL* paracentral lobule, *L* left, *R* right.

### Association between classification features and clinical variables

**Figure 2:**
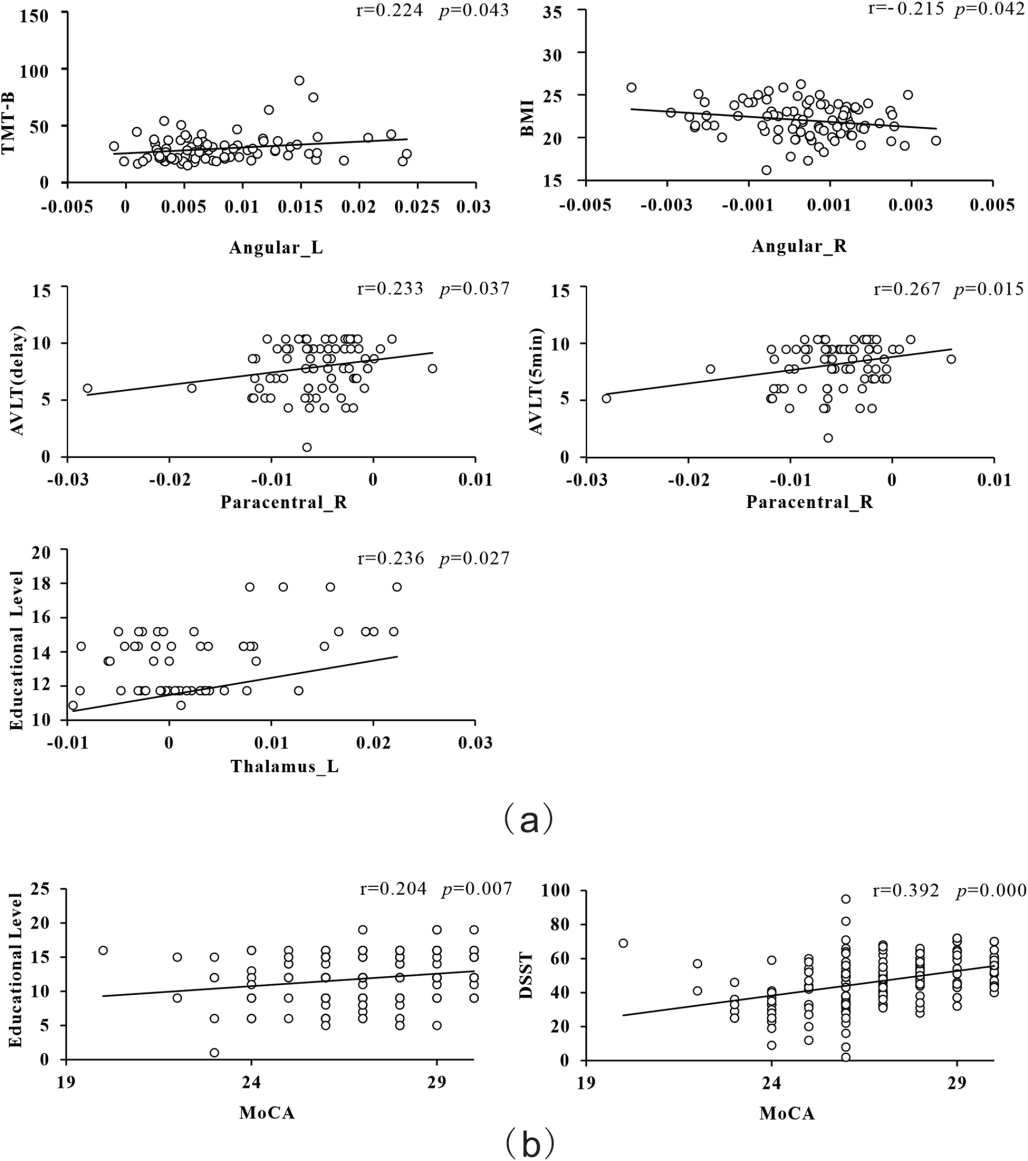
Associations between neuropsychological test scores and functional connectivity. Partial correlation was used to determine the relationship between neuropsychological test scores and functional connectivity. (**a**) Correlation of functional connectivity with neuropsychological test scores (**b**) Correlation between neuropsychological test scores. *TMT* trail-making test, *BMI* body mass index, *AVLT* World Health Organization University of California-Los Angeles auditory verbal learning test, *MoCA-B* montreal cognitive assessment-B, *DSST* digit symbol substitution test, *L* left, *R* right.

To better understand the relationship between the characteristics of the clinical development of T2DM-MCI, we further analyzed the correlation between imaging data and clinical variables (Bonferroni correction). The correlations between significant cognitive function scores and different brain regions were analyzed using Pearson’s correlation for three groups (Fig. 2). ANG.L was positively correlated with TMT-B (r = 0.224, *p* = 0.043), ANG.R is negatively correlated with BMI (r = –0.215, *p* = 0.042). PCL.R was positively correlated with AVLT (5 min) (r = 0.267, *p* = 0.015) and AVLT (delay) (r = 0.233, *p* = 0.037). THA.L was positively correlated with educational level (r = 0.236, *p* = 0.027). MoCA was positively correlated with DSST (r = 0.392, *p* = 0.000) and educational level (r = 0.204, *p* = 0.007). There was no significant correlation between other variables and the brain regions.

## Discussion

T2DM-MCI has a relatively low clinical diagnostic rate owing to its subtle onset and lack of clinical diagnostic approach. This study examined whether FC has discriminative features for accurately detecting T2DM-MCI using XGBoost patterns. The XGBoost algorithm is an accurate and efficient classification algorithm used in data mining with good performance. XGBoost has been applied for early diagnoses of diseases such as tuberculosis, epilepsy, kidney disease, and breast cancer^28–32^. Notably, this study is the first to apply whole-brain FC for detecting T2DM-MCI using the XGBoost model. Our model yields better classification performance (87.91% accuracy) than that of previous studies^21,22^. Using only 23 patients with T2DM and CI, Chen et al.^21^ used high-order FC for differentiating healthy controls from patients with T2DM and CI (79.17% accuracy) and patients with T2DM without CI (59.62% accuracy). With only 16 T2DM-MCI, Shi et al. employed large-scale FC to predict MoCA scores with a connectome-based predictive model and support vector machine, achieving AUC values (T2DM-NCI vs. T2DM-MCI) of 0.65‒0.70, which was significantly lower than that obtained by our method (0.82 in AUC). Moreover, our sample size was larger than those of previous studies^21,22,33^, including 199 participants in total.

T2DM is typically related to an increased risk of CI and dementia. Patients with T2DM may experience memory, language, attention, concentration, reaction, and executive function decline^1,34^. Nevertheless, researchers are still unsure of the exact pathophysiology underlying T2DM-related cognitive dysfunction, delaying the development of preventive treatments. We further found that the FC of THA, ANG, CAU, and PCL was highly discriminative in distinguishing T2DM-MCI, T2DM-NCI, and NC.

THA is a relay station or hub transmitting information between subcortical, cortical, and cerebellar areas^35^. THA declines with normal aging^36^. There may be no obvious structural damage, however, it develops thalamocortical FC impairment in patients with T2DM^21^. In our preliminary study^37^, patients with T2DM without CI already had abnormalities in the dynamic FC of THA, as revealed by a significant decrease in connectivity between the right executive control network and THA.L. Abnormal thalamic connectivity is associated with CI. Thalamic connectivity is likely to be impaired in patients with T2DM and CI, which is consistent with our results^21,38,39^. When undergoing external working memory tasks, the corresponding working memory brain regions are activated, and the right hippocampal/parahippocampal gyrus and THA are abnormally activated predominantly in the right cerebral hemisphere^40^. This indicates that THA is involved in processing working memory, and FC is already impaired before the onset of CI in patients with T2DM. ANG is associated with complex language functions and linked to other cognitive domains such as representational and semantic memory^41^. Patients with T2DM exhibit significantly thinner ANG cortical thickness^42^, reduced cerebral blood flow^43^, and less spontaneous neuronal activities^44^. Moreover, abnormal FC in THA and ANG because of diabetes causes various cognitive dysfunctions, including AD/VaD^38,45,46^. Compared with NC, bilateral ANG in patients with T2DM exhibit abnormal FC with multiple brain regions, and the FC of ANG with multiple brain regions positively correlated with MoCA, suggesting that the broad functional disconnectedness of ANG may play an essential role in the neuropathology of patients with T2DM-MCI^45^.

CAU is associated with memory and learning abilities^47^ as well as executive and cognitive processes^48^. CAU and the cerebellum function as a network that controls behavior^49^. FC between the CAU and hippocampus, which is an important anatomic basis for learning and memory, is implicated in altered white matter structure in patients with T2DM^50^. CAU has extensive connections to cortical and subcortical structures that serve complex regulation of motor function, cognition, and emotion^51^. In patients with T2DM, the grey matter volume of CAU is significantly reduced^42^, and the microstructure is abnormal^50^. The characteristics of the abnormalities are significantly associated with the duration of T2DM. In addition, the activation of the left CAU, hippocampus, and parahippocampal gyrus is weaker in T2DM-MCI than in normal controls under memory task stimulation^40^. Consistent with the previous studies mentioned above, the abnormality of FC of the CAU in our findings indicates impaired cognitive functions in patients with T2DM. In addition, PCL is associated with motor and sensory innervation of the contralateral lower extremities as well as the regulation of physiological functions. However, FC in PCL is affected in cognition-related diseases, such as vascular cognitive impairment. Sun et al. found that the most obvious regions showing connectivity deficits were between several regions, including PCL, and CAU.R^52^. They also showed impaired connectivity in the default mode network, and PCL with CAU.R. PCL was also discriminative as a region of interest (ROI) feature in the T2DM classification^33^. Furthermore, during the analysis of the internal connectivity of the left executive control network, ANG.L and PCL.L had significantly decreased connectivity with other brain regions in the network. In the external network connectivity analysis, significant differences were found between the left executive control network and ANG.R/PCL.L. In addition, significant differences were observed between the right executive control network and PCL.R/ANG.R. Furthermore, significant differences were found between the precuneus network and CAU.R/ANG^37^. In summary, THA, ANG, CAU, and PCL are highly sensitive to T2DM. They play essential roles in the early diagnosis of T2DM-MCI.

Educational level, age, BMI, blood pressure, and blood glucose levels are key factors influencing MCI in patients with T2DM. Correlation analysis showed that THA.L and MoCA were positively correlated with educational level, suggesting that highly-educated people have a lower risk of developing MCI^53,54^. Lower FC strength in ANG. R was associated with higher BMI. We also found that higher cognition scores were positively correlated with higher FC in PCL.R and ANG.L. This corresponds with previous findings^21,55^ that people with higher FC in PCL.R and ANG.L have a smaller risk of developing MCI.

Our study has some advantages. First, this study is the first to apply the XGBoost model to classify T2DM-MCI and achieve a good classification performance. Second, our analysis was based on whole-brain FC, unlike previous studies that were based on brain regions or predefined networks^8,21,56,57^. Third, we found that THA, ANG, CAU, and PCL demonstrated significant discriminative power in T2DM-MCI detection. However, our study has some limitations. First, the overall T2DM study sample was below 200; the number of T2DM-MCI was small. Therefore, multicenter data collection should be considered to expand the sample size in future studies. Second, this classification study extracted different characteristic connections based on all participants and applied the features to training classification, which has the problem of cross-validation and is slightly limited in the subsequent application. Subsequent studies can consider separating the training and test sets and conducting feature extraction so that the data results can be more objective and random. Third, our research is only a cross-sectional study. We believe that combining follow-up and longitudinal studies will better explain the mechanism of accelerated neurodegenerative changes in T2DM-MCI.

## Conclusion

This study proposes a novel framework to pool the connectivity features extracted from whole brain FC for detecting T2DM-MCI. The current study is the first attempt to use the XGBoost model to detect T2DM-MCI, which significantly enhances the prediction accuracy of the model. We show that the FC within THA, ANG, CAU, and PCL provides major information for detecting T2DM-MCI. Our results affirm that FC contains clinically relevant cognition-related information. Therefore, it is a potential biomarker for assessing the degree of cognitive decline. Overall, our findings provide valuable knowledge for classifying and predicting T2DM-related CI. These results have clinical implications in patients with T2DM. It can help in early clinical diagnosis and provides a basis for future studies.

## Methods

Two hundred and ten individuals were willing to join this study (May 10, 2021, to July 1, 2022). The exclusion criteria for the two groups were as follows: type 1 diabetes mellitus, impaired fasting glucose or impaired glucose tolerance^58^, hypertension, hypoglycemia (blood sugar levels < 3.9 mmol/L), hyperlipidemia, serious eye diseases (e.g., blindness), symptoms of neurological conditions (e.g., cerebral infarction or hemorrhage), history of neurological abnormality (e.g., Parkinson’s disease), severe head injuries or chronic head discomfort (e.g., migraine), BMI > 31 kg/m^2^, left- or mixed-handedness, substance (tobacco, alcohol, or psychoactive drug) abuse, taking medications that may affect cognition and memory within 6 months, specific abnormalities detected on conventional MRI scans or any other factors that may influence brain structure or function (e.g., extreme physical weakness, chronic infections, and other endocrine diseases). Patients with T2DM were diagnosed by two experienced endocrinologists following international clinical standards^59^. MCI was evaluated via Mini-Mental State Examination (MMSE) and MoCA-B (21 ≤ MoCA-B score < 26, and MMSE score > 24 were diagnosed with MCI)^60,61^.

Participants with brain tumors (n = 3), neuropsychiatric diseases (n = 4) (e.g., major depression or schizophrenia), or developmental disorders (n = 4) were excluded. Finally, 37 patients with T2DM-MCI, 93 patients with T2DM-NCI, and 69 NC were enrolled in this study. The source of patients with T2DM and NC corresponded with our previous study^37^. This study was approved by the ethics committee of The First Affiliated Hospital of Guangzhou University of Chinese Medicine (ID: NO. JY [2020] 288). Written informed consent was obtained from all participants. In addition, the study was conducted following approved guidelines.

### Demographic, clinical, and neuropsychological assessments

Demographic assessments include age, sex, educational level, past medical history, height, weight and medication history. Clinical assessments include HemoglobinA1c (HbA1c), C-Peptide, systolic blood pressure (SBP), diastolic blood pressure (DBP), total cholesterol (TC), triglyceride (TG), fasting serum insulin (FSI), low-density lipoprotein (LDL), fasting blood glucose (FBG), microalbuminuria (mALB), albumin/creatinine ratio (ACR), micro total protein (M-TP), 24-h urinary protein (24 h UPRO). Neuropsychological assessments include MoCA-B, MMSE, digit span test (DST), AVLT, TMT, GPT, and DSST which can be used to assess cognitive ability.

### MRI data acquisition

A Siemens (Munich, Germany) 3 T Prisma scanner with a standard 64-channel head coil was used to acquire fMRI imaging. All participants were placed in the supine position and tried their best to keep heads as still as possible while acquiring images. The detailed parameters of the multi-slice T2-weighted echo-planar imaging (EPI) sequence were as follows: TR = 2000 ms; TE = 30 ms; FOV = 100 mm; flip angle = 90°; matrix dimensions = 64 × 64; slice thickness = 3.5 mm; and number of slices = 33. Three-dimensional T1-weighted images were acquired with the following parameters: TR = 2,530 ms; TE = 2.98 ms; FOV = 256 × 224 mm^2^; inversion time = 1,100 ms; flip angle = 7°; matrix size = 224 × 256;, sagittal slices = 192; slice thickness = 1.0 mm; and voxel size = 0.5 × 0.5 × 1 mm^3^. The BOLD-fMRI gradient EPI sequence acquisition parameters were as follows: TR = 500 ms; TE = 30 ms; matrix dimensions = 64 × 64; FOV = 244 mm × 244 mm; slices thickness = 3.5 mm; voxel size = 3.5 mm × 3.5 mm; number of slices = 960, and scan time = 8 min.

### MRI image pre-processing

For fMRI data, the pre-processing was performed using SPM12 (Wellcome Department of Imaging Neurosciences, University College London, UK, http://www.fil.ion.ucl.ac.uk/spm), and the statistical analyses of imaging data were performed using GRETNA (GRETNA v2.0) in Matlab R2021b. First, the first 10-time point-scanned images were removed owing to the instability of the magnetic field at the beginning of the scan. Second, all functional images were realigned to the first image to correct head movement. All participants met the criteria of < 2 mm translation and < 2° rotation in any direction. Otherwise, their data were excluded. Third, the functional images were normalized to the MNI space using DARTEL and resampled to a 3 × 3 × 3 mm^3^ voxel size^62^. Fourth, we used an anisotropic 6-mm full-width half-maximum Gaussian kernel^63^ for spatial smoothing of the obtained images. Fifth, we detrended and removed linear trends. Sixth, we removed covariates, excluding white matter, grey matter, and cerebrospinal fluid influences. Seventh, 0.01‒0.08 Hz bandpass filtering was used to remove high and low-frequency signals. Eighth, we removed the FD_Threshold > 0.5 mm time points by “scrubbing” 1-time point before and 2-time points after. In summary, the pre-processing procedures included slice timing correction, realignment, normalization, smoothing, detrending, filtering, and scrubbing.

### Statistical analyses

All statistical analyses were performed using the SPSS software package (version 26.0). The measurement data of each group were described by mean ± standard deviation. The demographic, clinical, and neuropsychological assessment scores of the three groups were compared using multiple independent sample ANOVA^64^. Categorical data were evaluated using Chi-square analysis. Paired-sample t-tests were used for pre-and post-treatment intragroup comparisons. In addition, a partial test was used to examine the relationship between imaging indices, cognitive tests, and clinical data. *p* < 0.05 was used as the statistical significance level.

### Classification based on the ANOVA-XGBoost model

#### Feature abstraction based on FC network

The pre-processed fMRI BOLD data has dimensions of 950 × 90, where 950 denotes the number of time points in each fMRI scan, and 90 means the number of ROI derived from the Automated Anatomical Labelling atlas. We calculated the mean BOLD signals for each brain ROI by averaging the time series over all voxels within the ROI. Subsequently, based on the pre-processed fMRI data, we used the Pearson correlation coefficient^65^ to build an FC network for each participant in a matrix size of 90 × 90. Every node represented a brain ROI, and every edge measured the linear correlation between any pair of ROI.

Subsequently, we flattened the upper triangle elements of FC, thereby deriving a 4,005-dimensional [(90 × 90–90)/2] vector for each participant. However, these features may be redundant for classifying participants into the three experimental groups. Therefore, we applied ANOVA and Bonferroni correction analysis to extract features showing significant differences (*p* < 0.05) among the three groups. Finally, we generated 511 discriminative features for further classification.

#### Illustration of XGBoost model

**Figure 3:**
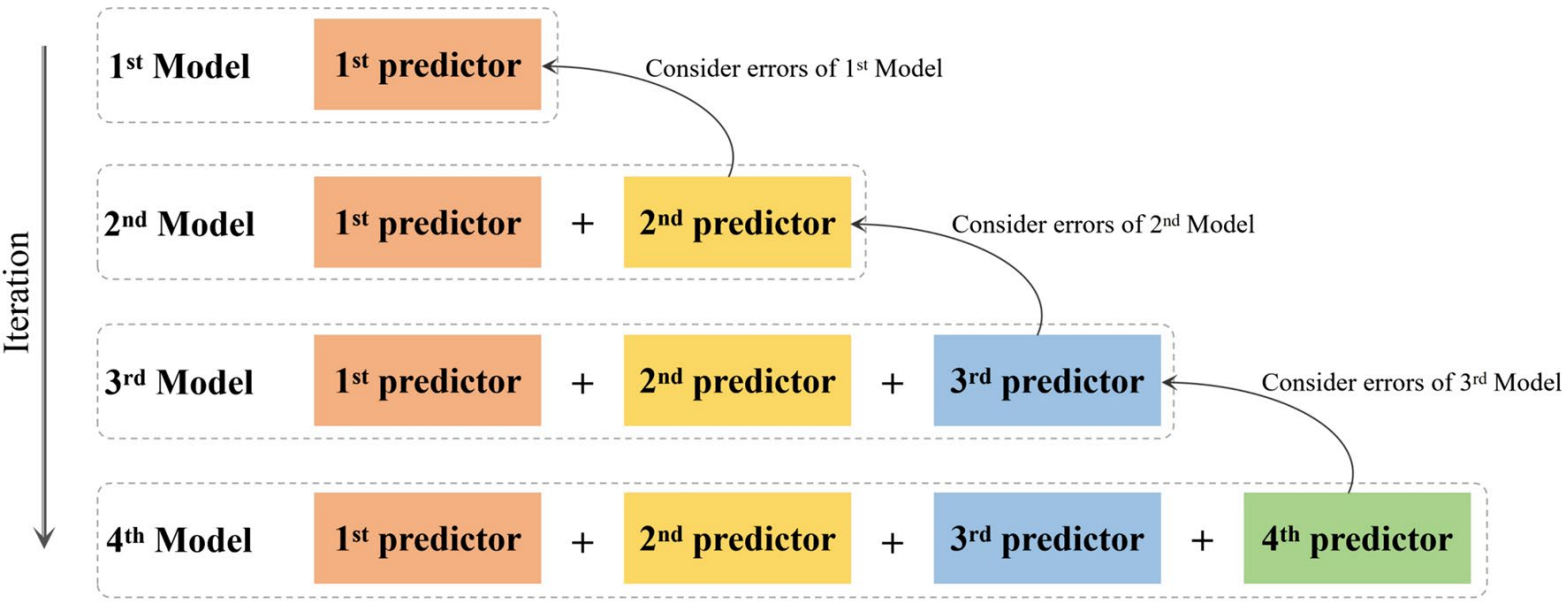
The illustration of XGBoost model for image classification. A predictor is trained to predict the errors made by the original model, and then construct an improved model whose output is fine-tuned based on the original prediction.

The XGBoost model^23^ is an ensemble machine learning algorithm based on decision trees that used the gradient boosting framework with promising performance in fMRI-related classification tasks^29,66,67^. The XGBoost model is illustrated in Fig. 3. The XGBoost model was built based on gradient boosting machines which used Gradient Boosting Trees^68^ as the error predictor. In gradient boosting, we trained a predictor to predict the errors made by the original model and constructed an improved model whose output was fine-tuned based on the original prediction. The improved model is an ensemble of two predictors, i.e., the original and error predictors. We repeated this process until we achieved satisfactory prediction results.

We used the XGBoost model to classify the three experimental groups, and the parameters were defined as follows: the number of gradient-boosted trees was 280; the maximum tree depth of base learners was 2; the minimum sum of sample weight required in a child was 5; the minimum loss reduction needed to make a further partition on a leaf node was 0; the subsample ratio of the training sample was 0.8; the subsample ratio for each tree’s construction was 0.8; the boosting learning rate was 0.1; and the L1 regularization constraint was 0.01. The XGBoost model was implemented based on the XGBoost package in Python (Supplementary Information S1).

### Experimental setting

We randomly partitioned all participants from the three groups into training and testing sets, following a 2:1 ratio, and this procedure was repeated five times. In the data division, we ensured that the three classes in both sets were equally distributed to prevent data imbalance. Five measurement metrics were adopted for model evaluation, including the AUC, ACC, F1, SEN, SPE, and PRE^69^.

## Supplementary Information

Supplementary Information.

## Data Availability

The data that support the findings of this study are available from the coauthors, Jinjian Wu and Shijun Qiu, upon reasonable request.
